# A Bibliometric Insight of Genetic Factors in ASD: Emerging Trends and New Developments

**DOI:** 10.3390/brainsci11010033

**Published:** 2020-12-31

**Authors:** Kang Wang, Weicheng Duan, Yijie Duan, Yuxin Yu, Xiuyi Chen, Yinhui Xu, Haihong Chen, Hongzhi Huang, Bo Xiong

**Affiliations:** 1 Department of Forensic Medicine, Tongji Medical College, Huazhong University of Science and Technology, Wuhan 430030, China; wangkjustice@hust.edu.cn (K.W.); duanweic@hust.edu.cn (W.D.); duanyijie@hust.edu.cn (Y.D.); huang1995@hust.edu.cn (H.H.); 2 The First Clinical College, Tongji Medical College, Huazhong University of Science and Technology, Wuhan 430022, China; yuyuxin@hust.edu.cn (Y.Y.); xuyinhui@hust.edu.cn (Y.X.); 3 Key Laboratory of Environment and Health (HUST), Ministry of Education, School of Public Health, Tongji Medical College, Huazhong University of Science and Technology, Wuhan 430030, China; chenxiuyi@hust.edu.cn; 4 School of Health Policy & Management, Nanjing Medical University, Nanjing 211166, China; chenhaihong@njmu.edu.cn

**Keywords:** bibliometric, ASD, genetics, quantitative analysis, research frontiers

## Abstract

Autism spectrum disorder (ASD) cases have increased rapidly in recent decades, which is associated with various genetic abnormalities. To provide a better understanding of the genetic factors in ASD, we assessed the global scientific output of the related studies. A total of 2944 studies published between 1997 and 2018 were included by systematic retrieval from the Web of Science (WoS) database, whose scientific landscapes were drawn and the tendencies and research frontiers were explored through bibliometric methods. The United States has been acting as a leading explorer of the field worldwide in recent years. The rapid development of high-throughput technologies and bioinformatics transferred the research method from the traditional classic method to a big data-based pipeline. As a consequence, the focused research area and tendency were also changed, as the contribution of de novo mutations in ASD has been a research hotspot in the past several years and probably will remain one into the near future, which is consistent with the current opinions of the major etiology of ASD. Therefore, more attention and financial support should be paid to the deciphering of the de novo mutations in ASD. Meanwhile, the effective cooperation of multi-research centers and scientists in different fields should be advocated in the next step of scientific research undertaken.

## 1. Introduction

Autism spectrum disorder (ASD) is considered a multifactorial syndrome which is characterized by three core characteristics, including qualitative impairment in interests and activities, social interaction, and communication paired with repetitive behaviors and may be accompanied by multiple neurological symptoms, such as intellectual and developmental disability (IDD), communication, speech, and language disorder, attention deficit and hyperactivity disorder, and so on [1]. The prevalence of ASD, which affects more than 1% of children globally, has been steadily increasing in recent years [2,3]. In the United States, 78% of families with autistic children were reported to have health care expenditures for their child, where 34% spent more than 3% of their income [4]. Thus, the families will undergo heavy economic burdens once their offspring is diagnosed with ASD [5]. Besides that, a considerable burden has been added to the public health care system and the government by ASD patients throughout their life. For children under 5 years old, ASD was ranked among the 20 leading causes of disability. Among the 5–14 age group of children, ASD was listed as the fourth leading cause of disability among mental disorders [6,7]. Special education services and parental job absences are the largest cost components for families with ASD children. During adulthood, medical costs are even higher compared with children. Also, residential nursing, supportive living accommodation, and loss of individual productivity contribute to the high burden and costs [8].

The developmental trajectory of the nervous system can be affected by many potential causes at different areas and stages. Given the temporal and spatial complexity of the disorder, examples of pathogenic mechanisms including social deprivation, genetic and metabolic defects [9], immune dysregulation [10], nutritional factors [11], and toxic or environmental factors have been reported [12]. With an estimated heritability of up to 80–90%, genetic factors are a major cause of ASD [13,14]. A series of genetic studies, including cytogenetics, linkage associations, genome-wide association studies (GWAS), and whole genome or exome sequencing, revealed that the genetic architecture of ASD is complicated and heterogeneous [3,15]. With the development and application of next generation sequencing (NGS), whole genome sequencing (WGS), and whole exon sequencing (WES), progress has been made in understanding the genetic causes of ASD. Hundreds of candidate genes of ASDs have been identified, such as, *KMT5B*, *FMR1*, *NAA15*, and *CHD8*. [16,17,18].

In this study, we aimed to conduct a bibliometric network analysis on the genetic research on ASD, which involved an objective measurement in scientific literature evaluation and aggregating the opinions of multiple scholars working in the field to mitigate researcher bias in reviews of the scientific literature [19]. The main goal of this analysis is to understand the structure, the current state of the art, and the future directions of the genetic studies in the ASD literature through a scientometrics approach. Utilizing statistical and mathematical methods, this study would provide a quantitative and qualitative analysis of the related literatures which can give a general overview on genetic factors in ASD and help grasp the research frontiers for future development [20,21]. 

## 2. Materials and Methods

**Figure 1:**
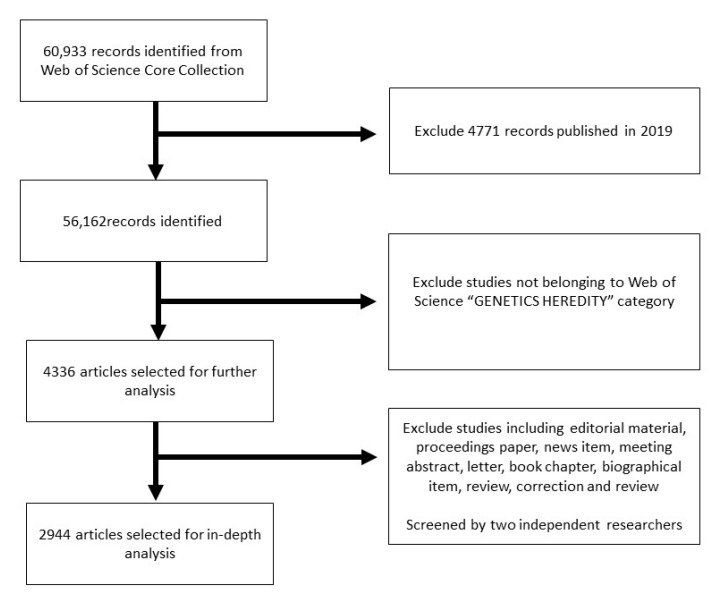
The flow chart of the methodology.

The Web of Science (WoS) database was used for searching the related literature. An initial database was built from WoSCC to retrieve the literature with the followingsearching strategy: ((Topic = (‘Autistic Disorder’) OR (‘Disorder, Autistic’) OR (‘Disorders, Autistic’) OR (‘Kanner’s Syndrome’) OR (‘Kanner Syndrome’) OR (‘Kanners Syndrome’) OR (‘Autism, Infantile’) OR (‘Infantile Autism’) OR (‘Autism’) OR (‘Autism, Early Infantile’) OR (‘Early Infantile Autism’) OR (‘Infantile Autism, Early’)). To ensure searching accuracy, the entry searching terms of autism were obtained from a standardized Medical Subject Headings (MeSH) list of searching terms from the National Library of Medicine. This search strategy identified 60,933 records over the whole previous year up to September 16, 2019. Next, we excluded 4771 records published in 2019, narrowed the retrieved literatures to the WoS genetics heredity category, and identified 4336 selected records for further analysis. To detect the original discoveries and minimize the bias as much as possible, we restricted the document types to original articles without restrictions based on language and finally obtained 2944 science literature examples for in-depth study and analysis (Figure 1). Raw data from the WoSCC were initially downloaded, checked, and verified by two independent investigators with different backgrounds. Any divergence was reconciled through discussion, and finally agreements were reached.

VOSViewer [22,23] 1.6.9 and CiteSpace [24,25] 5.5 were used to conduct statistical analyses of the scientific literature in genetic studies of ASD and to convert these references into visualized graphs. We drew the maps of annual and accumulated publication numbers, identified the contributions and collaborations between countries, performed co-citation analyses on references, and analyzed co-occurrence terms and burst keywords to detect the hotspots and frontiers.

## 3. Results

### 3.1. Analysis of Quantity and Growth Trend of Annual Publications

**Figure 2:**
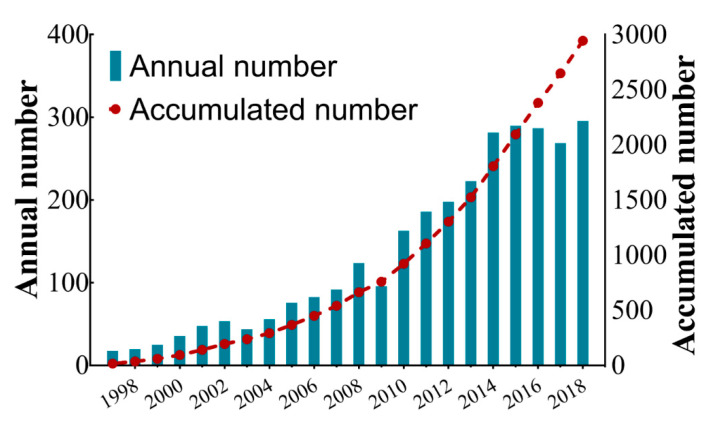
Accumulated number and annual number of genetic factors in autism spectrum disorder (ASD) publications from 1997 to 2018.

We performed a thorough search of the literature regarding genetic studies of ASD and retrieved 2944 items in total. The quantity of annually published studies was continuously increasing before 2014 and relatively stable from 2015 to 2018, with the average number of annually published literature as approximately 134 (Figure 2). In the 22-year period, the growth rate fluctuated, while the annual number of publications increased over time and the publication number increased nearly 17-fold from 17 records in 1997 to 295 in 2018.

### 3.2. Leading Countries and Institutions

**Figure 3:**
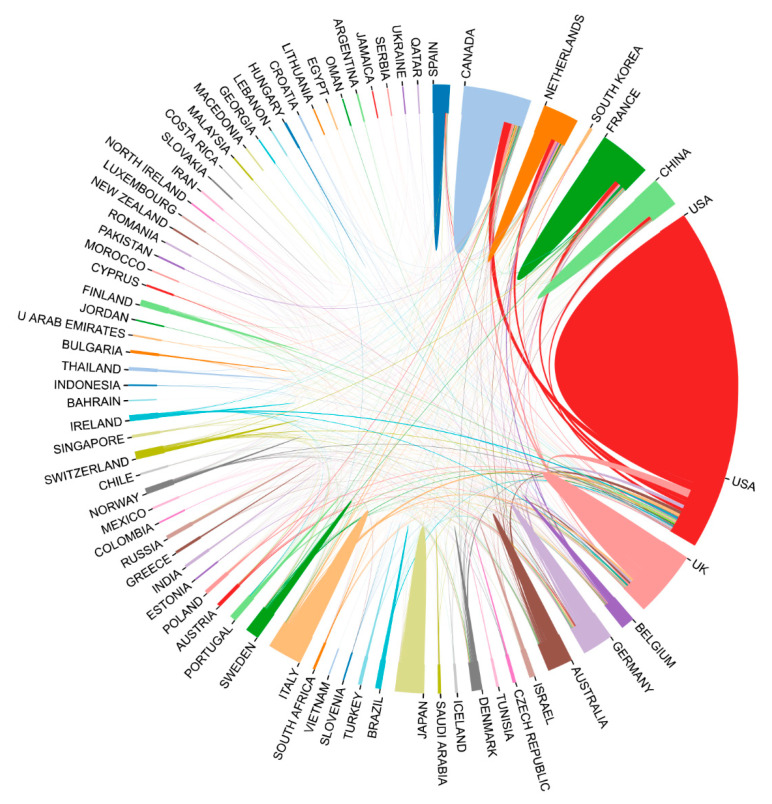
Cooperation between contributed countries.

**Figure 4:**
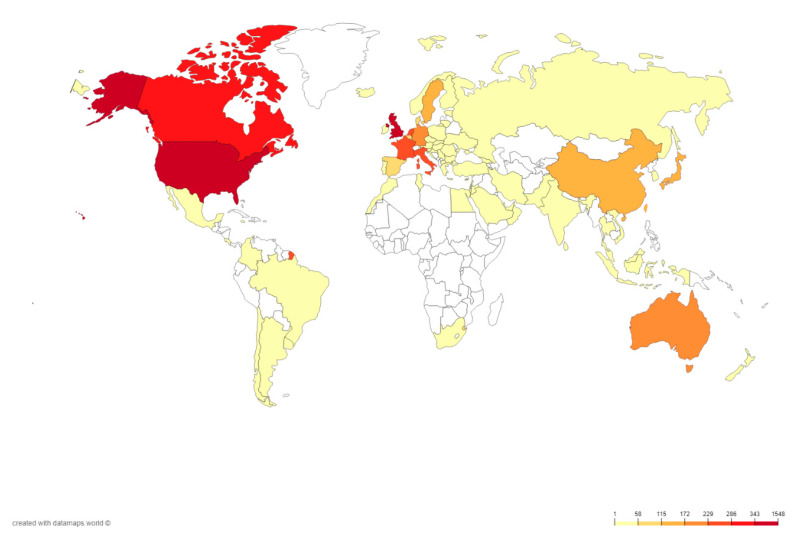
Geographical distribution of genetic factors in ASD publications.

We next evaluated the research activities regarding ASD genetics in different countries. The retrieved literature was contributed by over 70 countries, and an intensive cooperation pattern was clearly observed (Figure 3). The number of publications from a country or region is a sensitive indicator that reflects the attention placed and the research strength in the specific research area. In this regard, the United States participated in the greatest number of studies based on the results and maintained close cooperation with other countries with the highest H-index of 130, followed by England (77) and Canada (60) (Table 1). The average number of citations per article for publications in this field was 38.89, and the H-index was 145. The most effervescent research groups were mainly coming from North America and Europe. In addition, East Asia and Oceania contributed a number of research achievements (Figure 4). The top three countries of the citation/article ratios were Sweden (91.20), Germany (68.76), and the Netherlands (63.20). Furthermore, for Canada (55.283%), France (61.728%), and Sweden (58.197%), the major achievements were made by their core institutions. Nine of the top ten most productive countries were categorized as high-income countries, which produced approximately two-thirds (71.5%) of the documents in the field of genetic factors in ASD. These countries played a major role in ASD research and maintained a high degree of collaborations with other countries and regions.

### 3.3. Most Active Journals and Highly Cited Publications

Being important for exchanging, disseminating, and inheriting scientific findings, academic journals play key roles in the kingdom of science. According to our analyses, more than 120 scientific journals have published literature related to genetic studies of ASD. Based on Bradford’s distribution method [26,27], 3 core journals were highlighted (*Molecular Autism*, the *American Journal of Medical Genetics Part A*, and the *Journal of Intellectual Disability Research*), and the top 10 most productive journals were identified (Table 2). We retrieved the impact factor (2018), quartile, and Web of Science categories of these journals from the JCR database. Two of the three core journals were located in JCR quartile one, which are considered high-quality scientific publications in the JCR evaluation system. Different from the other core journals, the *Journal of Intellectual Disability Research*, which is an interdisciplinary journal combining biomedical research with social issues, was included in the social science citation index (SSCI) database. As for the top 10 highly cited articles (Table 3), they were all published in two top-notch journals: *Nature Genetics* and the *American Journal of Human Genetics*, which both had a relatively high impact factor compared with other scientific journals in the field. Interestingly, all top 10 highly cited articles came from the United States, which was consistent with its contribution in the field.

### 3.4. Development Skeleton and Scientific Landscapes of Genetic Factors in Autism

**Figure 5:**
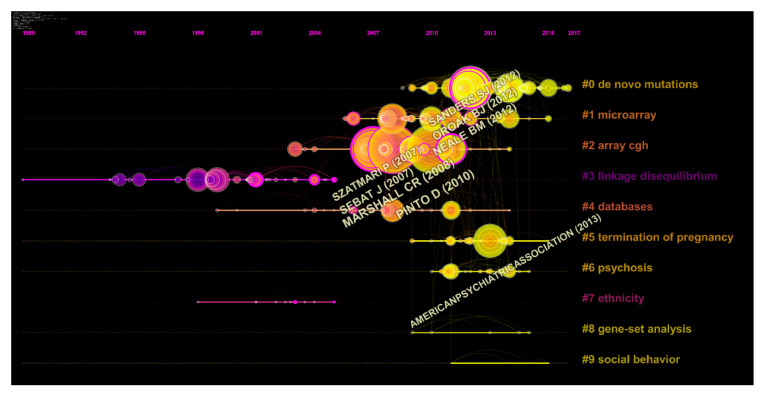
Co-cited references timeline map based on CiteSpace. Co-cited references that are commonly cited in the literature of genetic factors in ASD are clustered and identified by CiteSpace. Nodes on the map represent referenced documents. The shift of research concerns was reflected by the trend of co-cited literature subjects. Years are arranged horizontally at the top, and the label of each cluster is shown at the end of the cluster’s timeline.

The co-citation knowledge map refers to a network of co-citation publications, which is defined as the frequency at which two documents are cited together. Clusters of co-cited documents provide insights into the specialty structure of knowledge [38]. Therefore, we performed co-citation analysis to detect the literature with high co-citation frequencies in genetic studies of ASD to draw scientific landscapes, as well as to explore the historical development trends and correlation between the literature. According to the results of co-citation analysis, the documents could be clustered into 10 main groups, and a timeline map was generated by CiteSpace (Figure 5). The map indicated that most clusters were concentrated in the period from 2007 to 2016, namely in the latest decade. The earlier studies were mainly devoted to linkage disequilibrium (#3) and ethnicity (#7). Recent research focuses were shifted to de novo mutations (#0), microarray (#1), array comparative genome hybridization (CGH) (#2), termination of pregnancy (#5), gene set analysis (#8), and social behavior (#9), which are involved in the etiology and the intervention of ASD. As one of the most important achievements and powerful tools, studies on the database (#4) had been carried out during the time. Due to the genetic complexity and diversity among different countries, regions, and ethnicities, various databases were built to document the complex causes of ASD.

**Figure 6:**
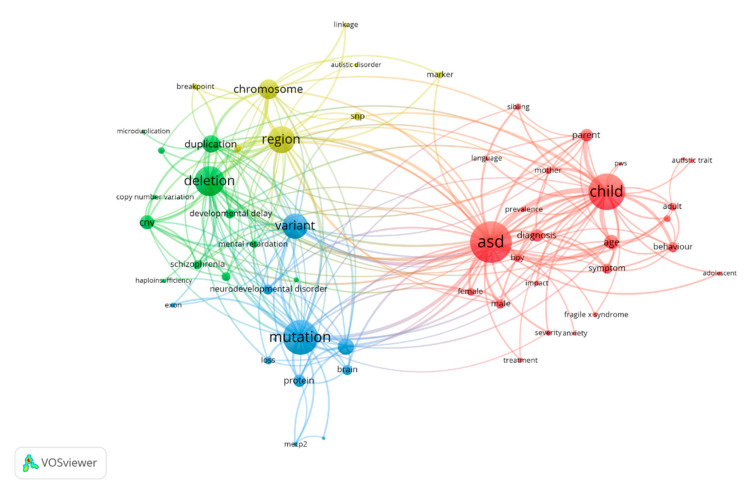
Co-occurrence network of terms of genetic factors in ASD publications from 1997 to 2018. The most common terms used in the scientific literature were investigated to identify relationships between the extracted terms and new aspects and the applied technologies. Terms were automatically exacted from titles and abstracts and divided into four clusters by the natural language processing techniques of VOSviewer.

We then performed bibliometric mapping with the VOSviewer software after excluding repeated and irrelevant items (Figure 6). Through the bibliometric analysis, the terms were automatically divided into four clusters. The red cluster was the largest group, whose terms were more focused on the common clinical symptoms and characteristics of ASD. The distinct clinical heterogeneity, gender, genetics, and comorbidity are recognized as the contributing factors. Clusters in green, blue, and yellow were tightly linked to each other due to their closer physical distance on the graph and were mainly related to ASD-related genetic abnormities, including single nucleotide polymorphisms (SNPs), biomarkers, deletion, or copy number variation (CNVs). The cluster in blue mainly includes the terms associated with mutations. The terms including CNVs, deletion, and duplication were gathered in the green cluster, and the cluster in yellow mainly covered the terms related to chromosome linkage analysis or SNPs.

### 3.5. Visualization Analysis of Focus Transfer and Research Frontiers

**Figure 7:**
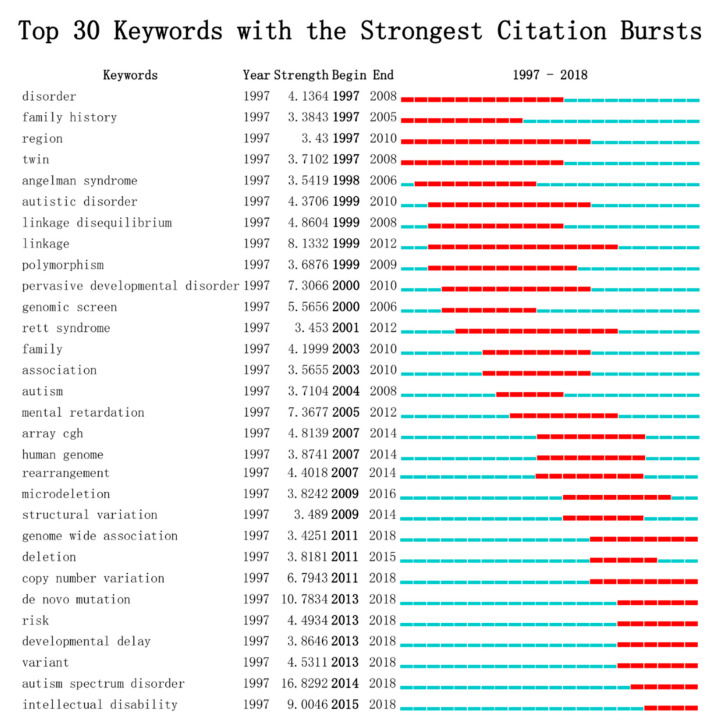
Top 30 keywords with the strongest citation bursts. Keywords with the strongest citation bursts in the scientific literature were analyzed and visualized in the keyword’s burst map. Each short line represents a year, and a line in red stands for the burst detection years. Keywords with red lines extending to the latest year can indicate the research frontiers a short period of time in the future.

To obtain the frontier topics and research tendency, keywords with the strongest citation bursts in the scientific literature were analyzed and visualized in a keywords burst map by the CiteSpace algorithm-dependent analytical tool, aligned based on their time of appearance (Figure 7). The keywords burst map contained two key points: the strength and beginning or ending year of the burst. The former represented the intensity of the burst, and the latter not only included the duration of the burst time but also revealed the transfer of research focus. Thirty keywords with the strongest citation bursts were included. Before 2000, burst keywords were mainly related to classical research techniques such as twin study (beginning in 1997) and family history study (beginning in 1997). Then, the genomic screen technique (beginning in 2000) and array comparative genome hybridization (array CGH) (beginning in 2007) were gradually developed and extensively applied in the first decade of the twenty-first century. In the latest decade, genome-wide association study (GWAS) (beginning in 2011) started to emerge. As for focused areas, researchers transferred from linkage disequilibrium phenomenon (beginning in 1999) to microdeletion (beginning in 2009) and structural variation (beginning in 2009), and the duration was eight years and six years, respectively. Nowadays, copy number variation (beginning in 2011) and de novo mutation (beginning in 2013) have become the most popular research topics. It was noteworthy that the keyword autism began in 2004 and ended in 2008 with a duration of 5 years, while the keyword autism spectrum disorder appears on the map and continues to the present, which might indicate that the studies were no longer focused on the specific neurological disorder autism, but a set of disorders with the umbrella term autism spectrum disorder. This transformation revealed a deeper understanding and notable progress on the study of ASD.

## 4. Discussion

In this study, we performed a systematic bibliometric assessment of the literature regarding genetic studies of ASD from 1997 to 2018. Previous related studies were mostly focused on ASD rather than its genetic factors and limited publication sources to only one specific country such as Spain, Brazil, or the United States [39,40,41]. One of the previous studies pointed out that the genetic fields got the most attention and received high numbers of citations within all ASD aspects [42]. Here, we aimed to obtain the landscape overview and the variation trend of the literature regarding the genetic factors of ASD. According to the results, the accumulated publication number had been growing steadily since 1997, when the first such literature appeared. Compared with the lower publication growth of research in forensic anthropology, the increase rate exhibited a relatively higher level, which indicated the importance of this field [43]. However, the annual publication number has not clearly increased in the last five years. The slow growth of financial investments or limited research positions might partially explain this. In terms of both the contribution number and publication quality, North America could be regarded as an undisputed leading region in this field, followed by European countries and two Asian countries, which was consistent with the medical big data research [44]. Interestingly, there were no publications from other countries except the United States on the top 10 most cited articles list, indicating that the US is leading ASD research. As a fast-developing country, China was listed on the top 10 productive countries. However, the H-index and citations per article were relatively low, indicating a requirement to improve research quality. Similar issues were also observed by bibliometric analysis in other areas, such as IL-35 research and hypertension [45,46].

To explore the branch development venation, the development of citation structures, size of clusters, and scientific landscapes over the past few decades were detected (Figure 5, Figure 6 and Figure 7). Sequencing technology and post-processing were leading a revolution in biological investigation due to the explosive development of biotechnology, data science tools, and interdisciplinary life science [47]. This led to the massive increase in databases that have been powerful resources for researchers, such as the autism genetic database [48], Simons Simplex Collection [49], and MSSNG [50]. More co-cited references have transferred from array CGH to microarray and finally to de novo mutations from 2007 to 2017 (Figure 5). Early studies clustered in array CGH and microarray mainly focused on CNVs, microdeletions, or microduplications. Many novel loci and ASD genes were identified, such as *SHANK2*, *ANKRD11*, and *DLGAP2* [31,51,52,53]. Finally, studies turned to de novo mutations. Various studies, which were performed using whole-exome sequencing or the multiplex targeted sequencing technique, revealed that de novo mutations were strongly associated with ASD [54,55]. These academic advances promoted genetic studies and pointed out new directions for researchers.

Using natural language processing techniques, VOSviewer extracts terms from the corpus file, where a term is defined as a sequence of nouns or noun phrases that can be found in a sentence. Over the past 22 years, deletion, variants, chromosomes, mutation, and duplication have become active research topics related to genetic factors of ASD. The four clusters displayed three major genetic research aspects and one major clinical aspect. CNVs, such as deletion or duplication, mainly affect the gene expression levels, which are supposed to be tightly related to developmental delay disease and schizophrenia [56,57]. Another research direction focused on gene mutations or variants. Several key ASD candidate genes were identified by targeted sequencing or WES to play an important role in the pathogenesis, such as *CHD8*, *WAC*, and *POGZ* [16,17,58,59]. There is no single biological marker, single gene, single region of the brain, or pathophysiological mechanism responsible for ASD [60]. Thus, multiple genetic factors were explored to give an account for it.

Different from the terms exacted from an article title or an abstract automatically, keywords are taken from the author-supplied keyword list of a publication. By defining some filed-in details and providing a common vocabulary and a tool with which the evolution of research can be studied, keywords play an important role in scientific research [61]. Burst keyword shifts imply the transformation of research focus and inspiration on research frontiers. The shifting of research hotspots implicates the transformation of the bias of fond assistance, and researchers should have put more spirit and mind to it so as to avoid detours in their careers. In the development of genome research, the appearance of omics is a watershed and of a significance that totally changed the research methods. However, we failed to catch the early research emphases that have not continued into the post-omics era because of the late start date. The keywords that burst until current day indicated the research frontiers and emerging trends of the publications of genetic factors in autism, including genome-wide association, copy number variation, developmental delay, intellectual disability, and so on. Autism spectrum disorder and de novo mutation were marked as the highest burst strengths along with them. Compared to the burst keyword autism appearing in 2004 and ending in 2008, the autism spectrum disorder burst from 2014 may indicate that the perception of autism transferred from a single disease to a kind of spectrum disorder. At the same time, researchers gained deeper insights into similar development disabilities, with developmental delay rising starting in 2013. With the appearance and rapid development of various high-throughput large-scale sequencing technologies, multiple studies have confirmed the contribution of de novo mutations of many single genes to the risk for autism spectrum disorders [54,62,63,64]. Transcriptomics studies underlining these high-risk gene knockouts in animals provided potential networks and possible transcriptional mechanisms for the etiology of ASD. Some of them also play a significant role in post-translational modifications or cellular energy metabolism. Their abnormalities can lead to neurodevelopmental disorders and cause psychiatric symptoms. Thus, it can be predicted that de novo mutations will continue to be a research focus in the near future. Finally, the linkage between ASD and intellectual disability has been brought to the attention of research communities since 2015. Increasing numbers of overlapping genes of ASD with other neurological disorders have been discovered recently, and the interactions of these genes and diseases indicate future interdisciplinary research directions. In summary, the burst keywords mainly reflected that the ASD research focused on genomic abnormalities. In comparison, the bibliometric myocardial infarction field mainly focused on microRNA with similar analyses [65].

## 5. Conclusions

We constructed a series of scientific maps of core journals, most contributions from countries or regions, co-cited references, and burst keywords to show the theme evolution and emerging trends of the ASD research field. As a highly heterogeneous disorder, a major ongoing research trend was to identify the de novo mutations related to the disease. Sequencing technology development and the era of omics led to the rapid growth of data volume. At this juncture, the field of ASD has the potential to draw inspiration and resources from breakthroughs in life science and IT technologies to make dramatic progress toward the understanding of the genetic neurobiology of brain science affecting children. Collaborations between scientists in different fields, such as clinical psychologists, data engineers, and brain scientists, will engender the expertise necessary for leveraging the power of large data sets to further our understanding of ASD.

## 6. Limitations

There were some limitations to this bibliometric work. First, the content of the science literature database is constantly changing from time to time, and numerous burgeoning scientific achievements were employed. Thus, the dataset on the published date should be broadened. However, compared with the original predominant dataset, its influence on the results was acceptable. Second, the *American Journal of Medical Genetics* had been divided into three journals—the *American Journal of Medical Genetics-part A*, the *American Journal of Medical Genetics-part B*, and the *American Journal of Medical Genetics-part C* by Wiley-Liss Inc.—several years ago. Here, we recognized them as four independent journals and calculated the published articles respectively. This method resulted in blank impact factors, SJR scores, and JCR quartiles of the *American Journal of Medical Genetics* in 2018 (Table 2).

## Figures and Tables

**Table 1:** Top countries and country institutions for autism genetics research.

Country	Articles	Citations	H-Index	Citations per Article	Top Country Institution	Top Institution Articles (%)
USA	1548	79,352	130	51.26	University of California System	304 (19.638%)
England	485	25,568	77	52.72	University of London	186 (38.351%)
Canada	315	17,817	60	56.56	University of Toronto	174 (55.283%)
Netherlands	250	15,799	56	63.20	Radboud University Nijmegen	98 (39.200%)
France	243	14,159	46	58.27	Institut National de La Sante et de La Recherche Medicale Inserm	150 (61.728%)
Italy	242	13,335	54	55.10	IRCCS Fondazione Stella Maris	32 (13.223%)
Germany	223	15,333	54	68.76	Helmholtz Association	52 (23.318%)
Australia	176	8247	45	46.86	University of Melbourne	39 (22.159%)
China	148	3169	29	21.41	Fudan University	22 (14.856%)
Sweden	122	11,126	40	91.20	Karolinska Institutet	71 (58.197%)

**Table 2:** The 10 most active journals that published articles on autism genetics research. SJR: SCImago Journal Rank.

Journal	Published Numbers (%)	IF 2018	SJR 2018	JCR Quartile	Categories
Mol Autism	373 (12.67%)	5.712	2.53	Q1	Genetics and Heredity; Neurosciences
Am J Med Genet A	304 (10.33%)	2.179	1.11	Q3	Genetics and Heredity
J Intell Disabil Res	282 (9.58%)	1.941	0.98	Q1	Education, Special (SSCI); Rehabilitation (SSCI)
Am J Med Genet B	192 (6.52%)	3.123	1.56	Q2	Genetics and Heredity; Psychiatry
Hum Mol Genet	176 (5.98%)	4.544	3.10	Q1	Genetics and Heredity; Biochemistry and Molecular Biology
Am J Hum Genet	137 (4.65%)	9.924	6.97	Q1	Genetics and Heredity
Eur J Hum Genet	114 (3.87%)	3.650	1.84	Q2	Genetics and Heredity; Biochemistry and Molecular Biology
J Med Genet	83 (2.82%)	5.899	3.02	Q1	Genetics and Heredity
Am J Med Genet	78 (2.65%)	NA	NA	NA	Genetics and Heredity
Eur J Med Genet	73 (2.48%)	2.022	0.90	Q3	Genetics and Heredity

**Table 3:** The characteristics of highly cited articles.

Rank	Total Citations	Article Title	Journal	Published Year	Country	IF 2018
1	2901	Rett syndrome is caused by mutations in X-linked MECP2, encoding methyl-CpG-binding protein 2 [28]	Nat Genet	1999	USA	25.455
2	1984	A general framework for estimating the relative pathogenicity of human genetic variants [29]	Nat Genet	2014	USA	25.455
3	1218	Consensus Statement: Chromosomal Microarray Is a First-Tier Clinical Diagnostic Test for Individuals with Developmental Disabilities or Congenital Anomalies [30]	Am J Hum Genet	2010	USA	9.924
4	1219	Structural variation of chromosomes in autism spectrum disorder [31]	Am J Hum Genet	2008	USA	9.924
5	1082	Mutations of the X-linked genes encoding neuroligins NLGN3 and NLGN4 are associated with autism [32]	Nat Genet	2003	USA	25.455
6	984	Genetic relationship between five psychiatric disorders estimated from genome-wide SNPs [33]	Nat Genet	2013	USA	25.455
7	925	Mapping autism risk loci using genetic linkage and chromosomal rearrangements [34]	Nat Genet	2007	USA	25.455
8	847	Mutations in the gene encoding the synaptic scaffolding protein SHANK3 are associated with autism spectrum disorders [35]	Nat Genet	2007	USA	25.455
9	703	A copy number variation morbidity map of developmental delay [36]	Nat Genet	2011	USA	25.455
10	692	Exome sequencing in sporadic autism spectrum disorders identifies severe de novo mutations [37]	Nat Genet	2011	USA	25.455

## Data Availability

No new data were created or analyzed in this study. Data sharing is not applicable to this article.
